# Correction: The relationship between glycaemic status and the risk of third, fourth and sixth cranial nerve palsy: a nationwide population-based study (2009–2018)

**DOI:** 10.1038/s41433-025-04160-y

**Published:** 2025-12-19

**Keywords:** Risk factors, Epidemiology

Correction to: *Eye* 10.1038/s41433-025-04011-w, published online 07 November 2025

The article “The relationship between glycaemic status and the risk of third, fourth and sixth cranial nerve palsy: a nationwide population-based study (2009–2018)”, written by Chaeyeon Lee, Kyung-Do Han, Juhwan Yoo et al., was originally published open access under a “Creative Commons Attribution (CC BY) license 4.0”. As a result of the author’s/authors’ subsequent decision not to publish this article under the open access model, the copyright notice of the article was changed on 19 November 2025 to © The Author(s), under exclusive licence to The Royal College of Ophthalmologists 2025 with all rights reserved.

The original article has been corrected.

